# Randomized Controlled Trial Evaluating the Benefit of a Novel Clinical Decision Support System for the Management of COVID-19 Patients in Home Quarantine: A Study Protocol

**DOI:** 10.3390/ijerph20032300

**Published:** 2023-01-28

**Authors:** Irene Alcoceba-Herrero, María Begoña Coco-Martín, Luis Leal-Vega, Adrián Martín-Gutiérrez, Lidia Peña-de Diego, Carlos Dueñas-Gutiérrez, Flor de Castro-Rodríguez, Pablo Royuela-Ruiz, Juan F. Arenillas-Lara

**Affiliations:** 1 Group of Applied Clinical Neurosciences and Advanced Data Analysis, Department of Medicine, Dermatology and Toxicology, University of Valladolid, 47005 Valladolid, Spain; 2 COVID-19 Unit, Department of Internal Medicine, University Clinical Hospital of Valladolid, 47003 Valladolid, Spain; 3 Emergency Medical Services (SEM) Direction, Sacyl, 47006 Valladolid, Spain; 4 Technical Direction of Primary Care, Sacyl, 47007 Valladolid, Spain; 5 Stroke Unit, Department of Neurology, University Clinical Hospital of Valladolid, 47003 Valladolid, Spain

**Keywords:** COVID-19, decision support system, Big Data, artificial intelligence, mobile health, telemedicine, wearable, monitoring

## Abstract

(1) Background: We present the protocol of a randomized controlled trial designed to evaluate the benefit of a novel clinical decision support system for the management of patients with COVID-19. (2) Methods: The study will recruit up to 500 participants (250 cases and 250 controls). Both groups will receive the conventional telephone follow-up protocol by primary care and will also be provided with access to a mobile application, in which they will be able to report their symptoms three times a day. In addition, patients in the active group will receive a wearable smartwatch and a pulse oximeter at home for real-time monitoring. The measured data will be visualized by primary care and emergency health service professionals, allowing them to detect in real time the progression and complications of the disease in order to promote early therapeutic interventions based on their clinical judgement. (3) Results: Ethical approval for this study was obtained from the Drug Research Ethics Committee of the Valladolid East Health Area (CASVE-NM-21-516). The results obtained from this study will form part of the thesis of two PhD students and will be disseminated through publication in a peer-reviewed journal. (4) Conclusions: The implementation of this telemonitoring system can be extrapolated to patients with other similar diseases, such as chronic diseases, with a high prevalence and need for close monitoring.

## 1. Introduction

In late December 2019, a number of cases of severe illness and death caused by Acute Respiratory Distress Syndrome (ARDS) were reported in Wuhan, China. The causative agent of these events was identified as Severe Acute Respiratory Syndrome Coronavirus 2 (SARS-CoV-2), which would later be responsible for the pandemic that has paralyzed the world for almost 2 years [1]. Since its outbreak, the coronavirus disease 2019 (COVID-19) has infected more than 640 million people and has resulted in 6.6 million deaths [2]. Spain has been one of the ten countries with the highest infection rate in the world; of a population of 47.5 million, around 13.5 million people were infected and 5000 individuals lost their lives due to the disease [2]. This high morbidity and mortality has been reduced with the vaccination of the population [2].

The most common symptoms of COVID-19 include fever, cough, headache, fatigue and gastrointestinal symptoms [3,4,5]. On the other hand, the main complication of the disease is ARDS, which appears as a consequence of the cytokine storm released in response to the infection. This condition is associated with a mortality ranging from 26% to 61.5% if admitted to a critical care setting and 65.7% to 94% in patients who received mechanical ventilation [6]. In this regard, the LUNG-SAFE study revealed that approximately 50% of mild ARDS goes undetected, with a probability of progression to moderate–severe ARDS of 40% and an in-hospital mortality rate of 30% [7,8]. Thus, the early detection of clinical worsening of COVID-19, manifested as ARDS or other potential serious complications, would allow for timely therapeutic interventions that would improve the health outcomes of these patients [9]. It should be noted that, in comparison with the first pandemic wave in early 2020, these figures have been significantly reduced by vaccination of the population, the emergence of variants with higher transmission but lower mortality rates and improved therapeutics for the disease [10].

As COVID-19 has a very broad clinical spectrum, the monitoring of the body temperature (BT), heart rate (HR), respiratory rate (RR), blood pressure (BP) and oxygen saturation (SatO2) may contribute to the early detection of clinical worsening or the occurrence of any major complications. For example, the risk of respiratory failure in the first 24 h after hospital admission based on the Quick COVID-19 Severity Index can be predicted by monitoring SatO2 and RR [11]. Similarly, alterations in certain vital signs such as BT (≥38 °C), RR (≥20 breaths per minute) and HR (>100 beats per minute) can help identify the existence of pneumonia among adult patients in primary care settings [12].

With this in mind, most studies within this line of research have used different devices to monitor each vital sign in isolation [13,14], with only a few assessing several parameters simultaneously. The latter includes one by Chung et al. [15] that used the HEARThermo device for continuous monitoring of BP and HR in patients with suspected or confirmed COVID-19 and another by Chinitz et al. [16] that used the Apple Watch Series 4 (Apple, Cupertino, CA, USA) to monitor arrhythmias and QT prolongation in COVID-19 patients in home quarantine. In addition, most of the devices that have been used for early detection of COVID-19 have been tested outside Europe [14,15,16,17,18].

One purpose of the protocol is to serve as a model for similar studies under similar conditions in order to improve pathology control. We propose to evaluate the benefit of a clinical decision support system based on: (i) the self-reporting of patient symptoms through a mobile application and (ii) the processing of data measured by wearable monitoring devices using artificial intelligence techniques in patients with COVID-19 in quarantine. All data generated will be uploaded to an online platform to which access will be provided to primary care and emergency health service professionals, allowing real-time detection of the progression of COVID-19 to severe forms and the occurrence of any potential major complications in order to promote early therapeutic interventions based on their clinical judgement. It is hypothesized that the implementation of the proposed clinical decision support system will improve the rate of detection of the clinical progression to severe forms of the disease in COVID-19 positive patients in home quarantine, compared to current practice.

The study will examine the differences between the active and control groups 30 days after the date of diagnosis of COVID-19, in terms of clinical progression of the disease to severe forms, defined by: progression to pneumonia, need for intensive care unit (ICU) admission, need for invasive mechanical ventilation and mortality rate. On the other hand, the secondary outcomes to be considered will be: the hospital admission rate, the average hospital stay, the occurrence of major vascular events (including acute myocardial infarction, stroke and vascular death), the economic cost of the derived care to the health system, the satisfaction of professionals and patients with the project and the usability of the technologies used in the project.

## 2. Materials and Methods

### 2.1. Study Design

Prospective, single-blind, multicenter, randomized clinical trial. Acute COVID-19 included study patients will be randomized to one of both study arms: conventional telephone calls from healthcare professionals, periodic telemonitoring with wearable devices and mobile phone application allowing self-reporting of symptoms three times a day versus traditional telephone calls from healthcare professionals and symptom reporting application.

### 2.2. Eligibility Criteria

To be considered for participation in the study, patients must meet all the inclusion criteria set out in this protocol without meeting any exclusion criteria (Table 1).

All patients participating in the study will receive an SMS message on their mobile phone with a link to download the healthcare app, from which they will initially electronically sign the informed consent form required to participate in the study (Supplementary Materials, File S1) and provide their contact details and home address for delivery of the Bakeey E66 smartwatch and Wellue FS20F pulse oximeter, if randomly assigned to the active group.

When patients are discharged and their home quarantine period ends, after seven or ten days depending on symptomatology, they will be able to remove the healthcare application called ‘‘Home app’’ from their mobile phone and stop wearing the monitoring devices, although their participation in the study will not end until six months after the date of the positive PCR or antigen test, when they will receive a final follow-up call from the data monitoring board to assess the primary and secondary outcome measures.

### 2.3. Setting

Patients will be recruited from the two health areas of Valladolid (total target population: 560,000 habitants). The primary care centers that have been selected by the Technical Direction of Primary Care in both areas are: from Valladolid East Health Area, the centers Barrio España, Cabezón de Pisuerga, Canterac, Cigales, Circunvalación, Gamazo, Iscar, Magdalena, Medina del Campo, Olmedo, Pilarica, Portillo, Renedo de Esgueva, Rondilla, San Isidro, Santovenia and Tudela de Duero; from the Valladolid West Health, the following centers: Area Arturo Eyries, Casa del Barco, Covaresa, Delicias, Huerta del Rey, La Cistérniga, Parquesol and Plaza del Ejército. Furthermore, the Health Emergency Service of Castilla y León will participate, managing and responding to the serious alerts produced by the platform.

### 2.4. Sampling

For the sample size calculation, the population of COVID-19 patients in the province of Valladolid during the 6-week period, November–December 2021 (3210 potential patients) is used as a frame. Conversely, for the calculation of the sampling error, the worst-case scenario of *p* and q = 0.50 and a confidence level of 1.96 was considered.

The consecutive sampling of all COVID-19 patients who meet the eligibility criteria will be conducted for a period of six weeks. The contact details of all patients who electronically sign the informed consent form required to participate will be recorded so that they can be contacted in six months for a follow-up call.

To ensure an equitable and randomized allocation of patients to one or the other study group, an automatic selection table will be used according to the number assigned to each patient in the study, consisting of three numerical digits indicating the consecutive order of inclusion (001, 002, 003, etc.). This selection table will be obtained by generating 500 random numbers so that each random number generated will be a case, and the rest will be a control. In this way, the process will end when 250 numbers (cases) are generated.

### 2.5. Study Overview

#### 2.5.1. Clinical Decision Support System

The clinical decision support system to be evaluated will supplement the conventional follow-up telephone call provided by primary care to COVID-19 patients (Supplementary Materials, File S2) and will consist of the following elements.

**Figure 1:**
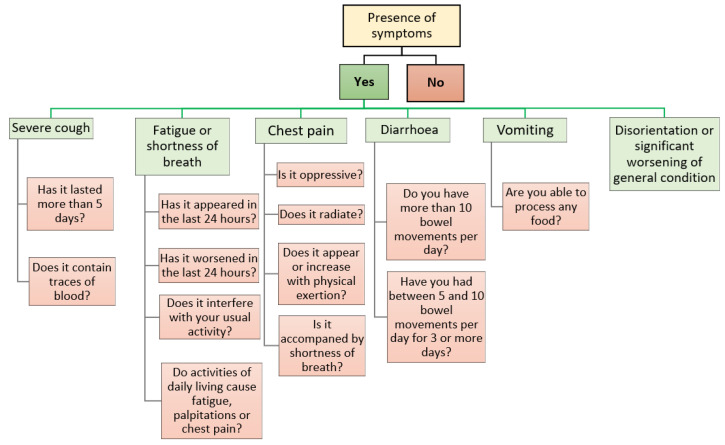
Symptoms and associated clinical questions covered by the ‘‘Home app’’.

The healthcare application called ‘‘Home app’’: all patients who meet the eligibility criteria and consent to participate will be provided with access to the ‘‘Home app’’, which they can download via SMS to their mobile phone. Importantly, this application will only be compatible with smartphones running an Android^®^ operating system (Google, Mountain View, CA, USA). The application will send push notifications to the patient three times a day (10:00 a.m., 4:00 p.m., 10:00 p.m.), asking them to indicate the presence of COVID-19 related symptoms and their characteristics, and will serve as a link for sending the vital signs data measured by the wearable monitoring devices to the server, where they will be further processed using artificial intelligence techniques (only in the active group). The symptomatology covered by the ‘‘Home app’’, together with the series of clinical questions associated with each symptom, are listed in Figure 1.

**Figure 2:**
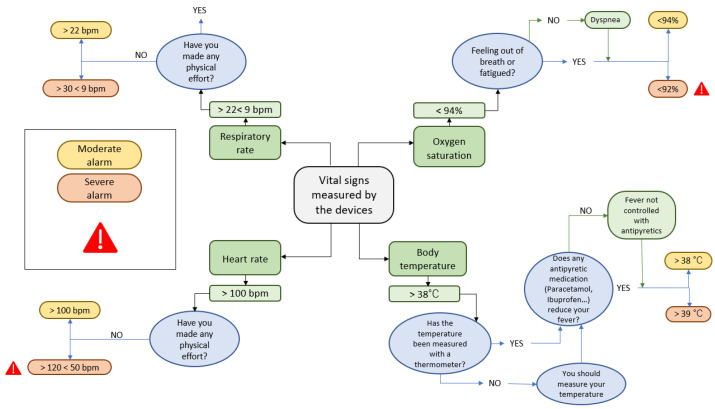
Decision tree for the generation of moderate or severe alerts based on vital signs monitored in patients allocated to the active group of the study.

Wearable monitoring devices: patients randomly assigned to the active group will receive, at home by express courier, the Bakeey E66 smartwatch (Shenzhen Yisi Technology Co. Ltd., Guangdong, China), for real-time monitoring of BT, HR and RR, and the FS20F Bluetooth Finger Oximeter (Wellue, Los Angeles, CA, USA), for self-reporting SatO2 values three times a day via the healthcare application (along with self-reporting of symptoms). Instructions will be provided to link participants, via Bluetooth, with the healthcare application and to send the monitored data to a server where it will be further processed using artificial intelligence techniques, generating moderate or severe alerts depending on whether they exceed certain pre-established values. Whenever an alarm is detected, a push notification will automatically be sent to the healthcare application, where the patient will have to answer a series of verification questions to confirm the veracity of the alarm (Figure 2).

**Figure 3:**
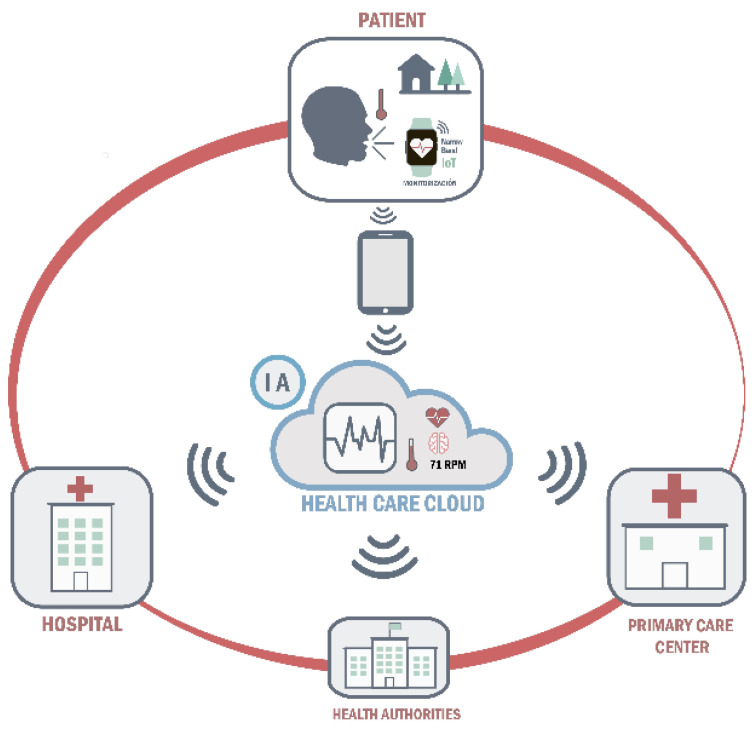
Clinical decision support system overview diagram.

Online platform: both the data measured by the wearable monitoring devices and the data self-reported by patients in the ‘‘Home app’’ will be uploaded to an online platform. and record that will be accessed daily by the primary care and emergency health service professionals who wish to participate in the study. Specifically, primary care professionals will be responsible for the management of moderate alarms and emergency health service professionals will be responsible for the management of severe alarms, being alerted of their existence through a computer-generated voice message. Participating healthcare professionals will have to register on the same platform and act in response to each alarm according to their clinical judgement. This will allow the research team to monitor the management of the different alarms generated throughout the study. In this way, the platform will not only serve as a clinical data registry to guide decision-making on the management of COVID-19 patients in home quarantine by both primary and specialized care, but will also serve as a repository of data that can be made available to public health agencies or even other researchers upon reasonable request (Figure 3).

Integration of the system modules: Regarding primary care, health professionals (nurses and doctors) will be responsible for recruiting patients. At first, the screening will be carried out on the online platform which will coincide with the first call of the conventional protocol, where it will be assessed if the patient meets the inclusion and exclusion criteria. If the patient meets the inclusion and exclusion criteria, the system will allow the baseline visit to be made, which will coincide with the first call after the patient is diagnosed with COVID-19. Data from the initial visit will be recorded, the new patient will be registered with an associated mobile phone number and an anonymous and unique ID will be provided. The online platform itself will randomly assign the patient to the case or control group. At that point, a message will be sent to activate the patient. Once the user opens the message, they will have to click on the link to download the health application called ‘‘Home app’’ where they will fill in their data, receive information about the project and data processing and sign the informed consent if they agree. In the case of the active group, a request will be sent, with the delivery address of the bracelet, to the transport company that previously arranged the shipment of the devices. Once the patient receives the bracelet, they will link the bracelet to the application and if they need support, they will receive it either via telephone call, which will be available for 14 h a day, or in the application itself. Healthcare professionals will be able to record on the web panel the registered telephone calls made according to the current protocol, the vital signs will be displayed in real-time, the responses that will be provided three times a day from patients on their perceived clinical state, and the alerts issued by the alteration of any parameter. In the event of a serious alarm, it will be received by the emergency system to provide immediate care, if required by the patient. At the end of the monitoring period, the primary health care staff will discharge the patient. In the case of the active group, the logistics will be activated to collect the devices and take them to a disinfection point. In addition, to improve the healthcare support system, a satisfaction survey and a usability survey will be carried out among patients and healthcare professionals who have participated in the study.

Patient and health professional support: the clinical decision support system (the application and wearable monitoring devices) is designed to make it easy for the patient to use, to promote patient autonomy, to make the user the center of the disease and to provide individualized health care. The application is very intuitive and the collection of vital signs will be carried out automatically, it is only necessary for the patient to have the devices in place. In case the patient needs technological support, a family member could, if desired, help to answer questions about the evolution of the development of the disease and manage the placement of the monitoring devices. In addition, patients will have access to a remote support system fourteen hours a day, via telephone contact or through an online chat in the application itself. The objective of providing remote support by qualified personnel is to promote greater patient and caregiver adherence. Furthermore, this support system can also be used by healthcare professionals to improve system management. It should be noted that the application will have a section of frequently asked questions, which will be updated and new ones will be added depending on the problems reported by patients and professionals, which will be answered employing infographics in very simple language, to favor the autonomy of the user and the healthcare professional.

Machine learning models will be used to implement the system and manage the data. To start with, the raw data from the wristbands will be calculated using the median of 5 min of monitoring constants, then new columns will be created to identify if there are anomalies, what type of alert it is (medical or technical), what type of parameter it measures and the level of alert (mild, moderate and severe). The selection of these columns will be based on clinical judgment and will define when an alert is sent. In order to generate predictions based on the best model to classify future patient alerts, different models will be implemented and the one that produces the least possible false positives and best fits the data flow of the system will be selected. In total, thirteen models will be implemented: Random Forest Classifier, Gradient Boosting Classifier, Ada Boost Classifier, Extra Trees Classifier, Decision Tree Classifier, Light Gradient Boosting Machine, K Neighbors Classifier, Naive Bayers, Quadratic Discriminant Analysis, Ridge Classifier, Linear Discriminant Analysis, Logistic Regression, SVM- Linear Kerner.

#### 2.5.2. Study Arms

Patients randomly assigned to the control group will receive the conventional telephone follow-up provided by primary care to COVID-19 patients and will also be able to report their symptoms 3 times a day via the healthcare application called ‘‘Home app’’, while patients randomly assigned to the active group will also receive the Bakeey E66 smartwatch and the Wellue SFS20 pulse oximeter at home. The only difference between patients who will be assigned to the experimental group and the management group is that the latter will not be provided with portable monitoring devices.

#### 2.5.3. Follow-Up Visit

Patient participation in the follow-up phase of the study will end when a second PCR or antigen test determines absence of virus. The final follow-up telephone visit to assess the primary and secondary outcome measures will be conducted on the 30th day after the date of the positive COVID-19 diagnosis. To minimize the risk of bias, primary and secondary endpoints will be assigned for analysis to an external endpoint adjudication committee. In addition, patients will be contacted by telephone after the end of the study to complete surveys on their satisfaction with participation in the study and usability of the ‘‘Home app’’ using the Spanish version of the System Usability Scale (SUS) [18].

#### 2.5.4. End of Study

Upon completion of the study, which is planned for 2022, a data monitoring board committee consisting of the research team and the coordinators of the primary care centers participating in the study will meet to distribute the satisfaction and usability surveys to the participating healthcare professionals and to collect the other primary and secondary outcome measures from the patients’ electronic health records. All these data will be made available for evaluation to an external blinded endpoint adjudication committee.

### 2.6. Patient and Public Involvement

Patients will actively participate in the study by reporting their symptoms three times a day and answering a series of verification questions when significant alterations in any of the monitored vital signs are detected (the latter only for patients in the active group). In addition, at the end of the study, both patients and participating healthcare professionals will be asked to complete a satisfaction and usability survey (SUS) to report on difficulties encountered with the Home app/online platform and areas for improvement.

### 2.7. Data Analyses Plan

A professional biostatistician has been consulted to design the data analysis plan for the study, in which descriptive analysis of all data will be provided, including means and SDs or medians for continuous variables and proportions for categorical variables. The recorded data will be processed statistically in an encrypted form.

The statistical analysis technique to be used will be the analysis of variance (ANOVA), which will allow determining the existence of significant differences between the two groups in terms of primary and secondary outcome measures. Also, the Kaplan–Meier estimator and Cox proportional hazards model will be used to establish possible predictors of mortality and/or ICU admission. The statistical software to be used will be Stata 15 and a *p*-value of <0.05 will be assumed for statistical significance.

To assess the economic impact of the intervention on the health and social system, a cost-utility analysis will be carried out to evaluate whether there are fewer hospitalizations and calls to the emergency health service in the active group than in the control group, which translates into lower costs associated with patient care. With this in mind, an acceptability curve analysis will be carried out to see whether the proposed clinical decision support system is more appropriate than the conventional primary care telephone follow-up in terms of cost-benefit ratio.

## 3. Results

As it is a protocol, this study has no results, but its publication will allow the development in the future of new lines of research based on this system for clinical decision-making, to provide personalized patient care, early detection of complications and better monitoring of the evolution of diseases. For all these reasons, we truly believe in the potential of its use for COVID-19, which could be applied in possible new variants that appear more severe, in future pandemics, in infectious diseases that require home isolation or for monitoring chronic pathologies that require a high degree of follow-up.

Ethics and Dissemination

This study follows the principles of the Declaration of Helsinki and was approved in its first version by the Drug Research Ethics Committee of the Valladolid East Health Area on 18 February 2021 (CASVE-NM-21-508). This board will be informed of any changes to this protocol and of any adverse events that may occur during the course of the research in order to consider termination of the study, if necessary.

The information obtained from the healthcare application and the wearable monitoring devices will be shared electronically with the research team for evaluation and analysis, respecting the privacy of the participants in accordance with the Organic Law 3/2018, of December 5, on Protection of Personal Data and Guarantee of Digital Rights. Participating patients may withdraw from the study at any time without affecting their usual relationship with their primary care team, although data collected up to the date of withdrawal may be used strictly for statistical analysis purposes.

All data will be assigned an identifying code and the accompanying name will be removed so that patients cannot be identified. This data will be stored in an electronic database that can only be accessed by the research team and clinical staff participating in the study with a specific username and password, ensuring all cybersecurity requirements, according to the office of the Public Information Security Service of the Regional Management of Castilla y León, to protect data transmitted over the Internet to preserve patient privacy. The information collected and stored will be anonymized through this information system and will be for the exclusive use of health professionals and the research team. The only data that will not be anonymous is the home addresses of participants, so that the delivery company can transport the monitoring devices, but they will not be able to access more information than that. In the case of a severe alert, the specific data for direct urgent health intervention will not be anonymized or pseudonymized for the specific patient exclusively for the emergency professionals managing the severe alert.

As a dissemination plan, results obtained from the study will be presented at national and international conferences and through peer-reviewed publications. Furthermore, two PhD students will publish and defend dissertations related to the study.

## 4. Discussion

This protocol on the clinical decision support system to be evaluated will serve as a link between primary care, specialized care and emergency health services, which will allow for more comprehensive and individualized care for patients with a positive diagnosis of COVID-19. In addition, this individualized and continuous health care system will allow the early detection of worsening disease and the identification of complications, such as ARDS, or episodes caused by hypercoagulability, such as venous thromboembolism, which occurred in one in ten hospitalized patients prior to vaccination of the population [19,20]. The system will allow early treatment by identifying disease progression, and thereby minimize short- and long-term sequelae [20]. The literature shows that during the five days before worsening, patients presented an increase in RR, a decrease in Sp02 and an increase in HR for the previous three days [21]. Similarly, the risk of respiratory failure in the first 24 h of admission can be identified using the Quick COVID-19 Severity Index, with the measurement of three basic parameters: Sp02, RR and fraction of inspired oxygen [19].

In the aftermath of the pandemic, numerous surveillance systems have been developed [22]. This has been performed using data extracted from mobile phones or portable biosensors, monitoring physiological parameters for early detection of worsening in asymptomatic patients who have had contact with a positive patient and were in home quarantine [16,22]. Contact tracing systems, initially used to monitor diseases such as Zika or Dengue, have also been perfected [23,24]. Similarly, systems have been implemented to reduce the spread of COVID-19 by monitoring contacts through code scanning, Global Positioning Systems (GPS) or Bluetooth [25,26,27].

On the other hand, most studies of patients with COVID-19 used a device to measure each variable in isolation [13]. This is the case of the study with the HEARThermo device that monitored real-time temperature for early detection and a follow-up for suspected COVID-19 patients [14,28]. The technology applied to healthcare is constantly developing, and sensor monitoring systems must become more and more convenient and less intrusive [29]. The meta-analysis by Htun et al. concludes that the best way to detect pneumonia is by monitoring vital signs, given the difficulty of performing a physical examination or chest X-ray; it establishes as clinical predictor parameters a RR ≥ 20 breaths per minute, a BT ≥ 38 °C, a HR > 100 beats per minute and crackles obtained by stethoscope, which we cannot measure in the present study. As can be inferred from the above, there are few clinically validated devices that can be used comfortably and reliably by the user. As primary characteristics, the aim is to use devices that are comfortable, reliable, low-cost and encompass the monitoring of as many variables as possible [30]. To this end, the Bakeey E66 smartwatch was validated with patients hospitalized by COVID-19 and it was concluded that it is reliable for the non-invasive multimodal monitoring of BT, HR and RR.

As a result of these issues, eHealth is constantly developing for the detection, diagnosis and monitoring of diseases, and real-time vital signs monitoring systems can have a fundamental impact on the development of health surveillance and therapeutic interventions [31]. This system will allow healthcare workers to support clinical decision making based on objective data such as vital signs measurement by the wearable, real-time symptoms reported by the patient, and the clinical evolution of patients. The scientific literature shows that wearables are an effective tool that detects alterations in vital signs that complement the symptoms reported by the patient; this system will be key to identifying clinical deterioration of different diseases such as COVID-19 [21].

This device will also allow the detection of alarm symptoms in real time and trigger a health alert for early attention in case of clinical progression of the disease or the appearance of a serious complication. Similar monitoring systems can identify patients in need of further testing, prioritizing the use and allocation of resources [21,32]. In turn, the proposed system will provide clinical decision support to primary health care professionals, reducing complications, the length of hospital stays and the negative impact on short- and long-term health. The comprehensive and individualized monitoring of the most vulnerable patients with COVID-19, such as those over 60 years of age, people with risk factors such as hypertension, diabetes, cardiovascular disease or cancer, will be possible [32].

Morbidity and mortality and the complications of the COVID-19 disease have decreased exponentially due to the mass vaccination of the population, but the clinical benefits expected from the provision of this follow-up could be extrapolated to patients with other diseases requiring multimodal, continuous and non-invasive telemonitoring. For example, patients with analogous conditions with other diseases, such as people with chronic pathologies requiring comprehensive monitoring, after an acute event such as a stroke or for the monitoring of the most vulnerable individuals. This system would promote real-time health monitoring through virtual triage that classifies each patient according to their symptomatology and vital signs, so that interventions or treatments are more appropriate to the characteristics of each patient. In addition, the amount of data collected would help to study and combat current and future outbreaks, discover digital biomarkers, health indicators for diagnosis and quantification of health conditions.

In addition, the study aims to address the problem of geographical dispersion present in our community [33], with the understanding that technology should serve to accommodate and provide healthcare support to people living in both urban and rural areas. The main beneficiaries of this system will be patients living in rural areas with difficult access to health services or with risk factors for worsening COVID-19 due to coexisting conditions such as hypertension, diabetes or oncological processes.

Finally, in relation to the application of artificial intelligence to the system, different machine learning models will be applied to select the one that best performs the alert management, producing fewer false positives. A total of thirteen models will be applied: Random Forest Classifier, Gradient Boosting Classifier, Ada Boost Classifier, Extra Trees Classifier, Decision Tree Classifier, Light Gradient Boosting Machine, K Neighbors Classifier, Naive Bayers, Quadratic Discriminant Analysis, Ridge Classifier, Linear Discriminant Analysis, Logistic Regression, SVM- Linear Kerner. For the execution of this analysis, synthetic data similar to real patients will be generated and data with anomalies will be inserted. A sufficient number of samples will be generated so that the classification model has enough data and performs well when connected to the database of our wristbands. In preliminary tests, the best classification model so far has been Random Forest model.

Limitations

Some limitations of the protocol to be implemented are the need for an Android mobile operating system to download the healthcare application, the need for the patient to have digital skills to use the application (which may make it difficult to include elderly people) and the need to include a pulse oximetry device for oxygen saturation self-reporting in the active group.

Another important limitation is that the recruitment and results will be conditioned by the evolution of the pandemic, the emergence of new variants of the virus that behave differently from previous ones and, above all, because the study will be carried out in a population that is mostly vaccinated with two doses (80–85% of the city’s population will have two doses at the start of the study).

The protocol is designed for use in patients with COVID-19 requiring home isolation, which will be useful for other pandemics or infectious diseases requiring isolation to reduce the spread of disease but will limit its implementation when isolation does not apply.

## 5. Conclusions

This will be the first randomized clinical trial in Spain to examine the benefit of a clinical decision support system incorporating wearable monitoring devices for the management of COVID-19 patients. This system will be a link between primary care, specialized care and the emergency health service, allowing for individualized care, early detection of complications and the prevention of short- and long-term sequelae in COVID-19 patients.

The publication of this protocol will allow new lines of research to be developed in the future based on this telemonitoring model, in order to provide personalized patient care and the early detection of complications. The potential of this clinical decision support protocol lies in the role that new COVID-19 variants with more severe effects, infectious diseases requiring home isolation, or the emergence of other pandemics requiring the close monitoring and surveillance of worsening disease in patients may play. It can also be extrapolated to patients with other similar diseases, such as chronic diseases, with a high prevalence and need for close monitoring.

## Figures and Tables

**Table 1:** Eligibility criteria for participation in the study.

**Inclusion criteria**
- Positive diagnosis of COVID-19 by polymerase chain reaction (PCR) or antigen test. - Time window of ≤ 6 days between COVID-19 positive diagnosis and inclusion. - Internet access at home. - Possession of a smartphone with an Android^®^ operating system. - Willingness to electronically sign the informed consent form required to participate in the study and wear the monitoring devices if randomly assigned to the active group.
**Exclusion criteria**
- Age under 16 years. - Lack of digital skills to use the healthcare application called ‘‘Home app’’. - Possession of a smartphone with an iOS operating system. - Significant clinical progression that could lead to hospital admission between the date of positive COVID-19 diagnosis and the date of inclusion in the study. - Disabling upper limb pathology or cognitive impairment that may prevent participation in the study.

## Data Availability

Trial registration: NCT04802018. Registered 16 March 2021, https://clinicaltrials.gov/ct2/show/NCT04802018.

## Supplementary Materials

The following supporting information can be downloaded at: https://www.mdpi.com/article/10.3390/ijerph20032300/s1, File S1: Informed consent document for clinical research not involving biological samples; File S2: Protocol for the management and follow-up of acute and post-acute COVID-19 patients in primary care. Castilla y León, Spain
