# Supplementary material for: Randomized Controlled Trial Evaluating the Benefit of a Novel Clinical Decision Support System for the Management of COVID-19 Patients in Home Quarantine: A Study Protocol
Source: Int J Environ Res Public Health. 2023 Jan 28;20(3):2300. doi: 10.3390/ijerph20032300 (PMC9915322; doi:10.3390/ijerph20032300)
Supplement: Supplementary file 1 [file ijerph-20-02300-s001.zip › ijerph-2106399-supplementary.pdf]

File S1

Informed consent document for clinical research not involving biological samples.

HCUV HOSPITAL CLÍNICO UNIVERSITARIO DE VALLADOLID<br>Avda. Ramón y Cajal, 3 - 47003 Valladolid<br>Tel.: 983 42 00 00 - Fax 983 25 75 11<br>gerente.hcuv@saludcastillayleon.es	**INFORMED CONSENT DOCUMENT FOR CLINICAL RESEARCH NOT INVOLVING BIOLOGICAL SAMPLES**<br>**UNIVERSITY CLINICAL HOSPITAL OF VALLADOLID**	Sacyl<br>*V.ABRIL-2020*

INFORMED CONSENT DOCUMENT FOR CLINICAL RESEARCH NOT INVOLVINGBIOLOGICAL SAMPLES

UNIVERSITY CLINICAL HOSPITAL OF VALLADOLID

SERVICE: Neurology

RESPONSIBLE INVESTIGATOR: Juan F. Arenillas Lara

CONTACT TELEPHONE: +34637890926

EMAIL: jfarenillas@saludcastillayleon.es

NAME OF THE LINE OF WORK: SOY+ EXTRA-HOSPITAL PHASE

DOCUMENT VERSION: (Version number, date): 1.1, 20/04/2021

I) Purpose of the proposed line of work:

The present research study in which you are invited to participate aims to evaluate a clinical decision support system based on non-invasive multimodal out-of-hospital monitoring of COVID-19 positive patients in social isolation due to home confinement. If you meet the criteria for inclusion in the study, and consent to participate in the study, you will be randomly assigned to either the control group or the active group. Both groups will have to download the SOY+ application on their mobile phone, where they will be able to report their symptoms 3 times a day on a daily basis. In addition, the active group will be assigned two external monitoring devices, a pulse oximeter to measure oxygen saturation (SatO2) and a smart bracelet equipped with temperature (Tª), heart rate (HR) and respiratory rate (RF) sensors, whose data will be sent to health professionals in the Primary Care (PC) teams of the Valladolid East and West Health Areas, thusenabling early detection of the clinical progression of the disease to severe forms and/or the appearanceof significant events. Depending on the parameters determined by the devices, the system will produce mild or moderate alerts, which will be managed by the PC, and severe alerts, which will be managed by the Emergency Medical Service (EMS). Depending on the alerts generated, health professionals will be able to send health support to the home, make an additional phone call or carry out other therapeutic actions they deem appropriate based on

their clinical judgement. The main objective is to study whetherthere are significant differences between the follow-up of the control group and the active group in terms of the rate of clinical progression of the disease.

II) Some considerations about your participation:

A) Your participation in this study is completely voluntary. Within the process of hospital care for your illness, a clinical survey will be carried out, which will not interfere with the diagnostic or therapeutic processes that you have to receive due to your illness.

B) You may ask any questions you may have about your participation in this study.

C) You will not receive any financial or other compensation for your participation in the study. However, the information generated in this study could be a source of commercial benefit. If this is the case, mechanisms are in place to ensure that these benefits accrue to the health of the population, but not to you individually.

D) Personal data will be processed in accordance with the provisions of the applicable regulations, such as Regulation (EU) 2016/679, of 27 April, General Personal Data Protection, and its implementing regulations at both national and European level.

E) The information obtained will be collected by the data controller in an activity register and the SACYL and the University of Valladolid may have access to it for research or therapeutic purposes.

F) The data recorded will be processed statistically in a coded form. At any time, the participant will have the right to access, modify, oppose, rectify or cancel the data deposited in the database, provided that he/she expressly requests it. To do so, he/she must contact the principal investigator of the study. The data will remain under the responsibility of the Principal Investigator of the study, **Dr. Juan F. Arenillas Lara**. You also have the right to contact the Data Protection Agency if you are not satisfied.

G) The data will be kept indefinitely, which will allow them to be used by the principal investigator's group in future research studies related to the line of work described above. Said data may be transferred to other researchers designated by the Principal Investigator for work related to this line of work, always in the service of projects of high scientific quality and respect for ethical principles. In the latter two cases, authorization will be requested beforehand from the Drug Research Ethics Committee of Valladolid East Health Area.

Data controller	Valladolid Health Area Management
Purpose of the data	Use of personal data associated with the conduct of a clinical trialwith patients infected with COVID-19, specifically:<br>• Sending of App link for installation on Smartphone.<br>• Request for data to sign the informed consent form.<br>• Sending of the monitoring devices.<br>• Call by the SEM for critical alert.
Legitimation	Public interest associated with the control of the pandemic and its spread, according to recital 46 of the RGPD.
Recipients	University of Valladolid.

Rights	The interested party may contact the Valladolid Health Management to exercise their rights of access, rectification or deletion, limitation of processing or opposition to the processing of their clinical data. Data portability will not be carried out.
Origin	All data are provided by the interested party.

H) The lack of consent or the revocation of this previously granted consent shall not imply any detriment to the care you receive.

I) It is possible that the studies carried out may provide information relevant to your health or that of your relatives. You have the right to know this information and to pass it on to your relatives if you so wish.

J) Only if you wish, there is a possibility that you may be contacted in the future to complete or update the information associated with the study.

WRITTEN INFORMED CONSENT OF THE PATIENT

PILOT STUDY TO EVALUATE A CLINICAL DECISION SUPPORT SYSTEM BASED ON NON-INVASIVE MULTI- MODAL MONITORING OF COVID-19 PATIENTS IN SOCIAL ISOLATION DUE TO DOMICILE CONFINEMENT (SOY+).

I, _

____

____

(Name and surnames and surname of patient or legal representative)

I have read the information given to me.
I have received the information sheet that has been given to me. I have been able to ask questions about the study.
I have received sufficient information about the study.
I have discussed the study with

____

(Researcher's name and surname)

I understand that my participation is voluntary.
I understand that I can withdraw from the study:
1.- Whenever I want.
2.- Without having to give explanations.
3.- Without this having any repercussions on my medical care.

I hereby give my free and informed consent to participate in this research.

I agree that the doctors of the HOSPITAL CLÍNICO UNIVERSITARIO DE VALLADOLID may contact me in the future in the event that new data are needed. YES ☐ NO ☐ (mark with an X where appropriate)

I agree that the doctors of the HOSPITAL CLÍNICO UNIVERSITARIO DE VALLADOLID may contact me in the event that the studies carried out on my data provide information relevant to my health or that of my family members. YES ☐ NO ☐ (mark with an X where appropriate)

Once signed, I will be given a copy of the consent document.

____

SIGNATURE OF THE PATIENT / LEGAL REPRESENTATIVE NAME AND SURNAME DATE

IN THE OFFICE OF (Relationship, legal guardian, etc.):

I have fully explained the relevant details of this study to the patient named above and/or the person authorised to give consent on behalf of the patient.

____

SIGNATURE OF THE PRINCIPAL INVESTIGATOR NAME AND SURNAME DATE

SECTION FOR REVOCATION OF CONSENT (CONTACT PRINCIPAL INVESTIGATOR)

I ____ revoke the consent to participate in the study, signed above dated ________________________________

____

SIGNATURE OF THE PATIENT / LEGAL REPRESENTATIVE NAME AND SURNAME DATE

IN THE OFFICE OF (Relationship, legal guardian, etc.):

File S2

Protocol for the management and follow-up of acute and post-acute COVID-19 patients in primary care. Castilla y León, Spain.

TELEPHONE CLINICAL ASSESSMENT QUESTIONNAIRE IN THE HOME FOLLOW-UP OF PATIENTS WITH SARS-COV-2 INFECTION		
**Compared to the last time we contacted you, how are you feeling today?**<br>- Better<br>- Same<br>- Worse		
**QUESTION**	**YES**	**NO**
**Temperature**<br>- Fever<br>- More than 39ºC<br>- More than 3 days?		

**Dyspnea (even mild: NYHA I/II)**<br>- Do you feel "shortness of breath" or fatigue at any time?<br>- Do you have a slight limitation of physical activity?<br>- Does ordinary activity cause fatigue, palpitations or chest pain?<br>- Tachypnoea: slurred speech<br>- Severe cough for more than 5 days		
**Chest pain**<br>- On exertion<br>- Pleuritic type (rib pain that makes breathing difficult)		
**Hemoptysis**		
**Alertness** (interviewing the sick person and/or caregiver)<br>- Are you disoriented or confused?		
**Vomiting**<br>- Are they preventing feeding?		
**Diarrhea**<br>- More than 10 stools/day or 5-10/more than 3 days?		
**Inadequate social conditions**		
**Worsening of emotional state**		
**General non-specific malaise**<br>Maintain vigilance<br>If suspected silent hypoxia, measure SatO2		
